# Lack of association between oestrogen receptor polymorphisms and change in bone mineral density with tamoxifen therapy

**DOI:** 10.1038/sj.bjc.6605460

**Published:** 2009-12-01

**Authors:** N L Henry, A Nguyen, F Azzouz, L Li, J Robarge, S Philips, D Cao, T C Skaar, J M Rae, A M Storniolo, D A Flockhart, D F Hayes, V Stearns

**Affiliations:** 1 Breast Oncology Program, University of Michigan Comprehensive Cancer Center, Ann Arbor, MI 48109, USA; 2 Division of Clinical Pharmacology, Department of Medicine, Indiana University School of Medicine, Indianapolis, IN 46202, USA; 3 Breast Cancer Program, Sidney Kimmel Comprehensive Cancer Center, Johns Hopkins University, Baltimore, MD 21287, USA

**Keywords:** tamoxifen, bone mineral density, oestrogen receptor, CYP2D6, polymorphism, chemotherapy

## Abstract

**Background:**

Tamoxifen, a selective oestrogen receptor (ER) modulator, increases bone mineral density (BMD) in postmenopausal women and decreases BMD in premenopausal women. We hypothesised that inherited variants in candidate genes involved in oestrogen signalling and tamoxifen metabolism might be associated with tamoxifen effects in bone.

**Methods:**

A total of 297 women who were initiating tamoxifen therapy were enrolled in a prospective multicentre clinical trial. Lumbar spine and total hip BMD values were measured using dual-energy X-ray absorptiometry (DXA) at baseline and after 12 months of tamoxifen therapy. Single-nucleotide polymorphisms (SNPs) in *ESR1, ESR2*, and *CYP2D6* were tested for associations in the context of menopausal status and previous chemotherapy, with a mean percentage change in BMD over 12 months.

**Results:**

The percentage increase in BMD was greater in postmenopausal women and in those patients who had been treated with chemotherapy. No significant associations between tested SNPs and either baseline BMD or change in BMD with 1 year of tamoxifen therapy were detected.

**Conclusion:**

The evaluated SNPs in ESR and CYP2D6 do not seem to influence BMD in tamoxifen-treated subjects.

As mortality from breast cancer has been decreasing (Peto *et al*, 2000), delayed complications of therapy for breast cancer are becoming more apparent. In particular, osteoporosis is of substantial concern in breast cancer survivors, especially in those diagnosed while still premenopausal. Chemotherapy may induce ovarian failure in up to 70% of women (Petrek *et al*, 2006; Stearns *et al*, 2006), and the resulting oestrogen depletion can lead to bone loss (Saarto *et al*, 1997). In addition, chemotherapy can have direct detrimental effects on bone density through an inhibition of bone proliferation (Lester *et al*, 2005). It is well recognised that endocrine therapies for breast cancer can have variable effects on bone mineral density (BMD), depending on the pharmaceutical agent and the patient population (Chien and Goss, 2006; Perez *et al*, 2006; Coleman *et al*, 2007; Eastell *et al*, 2008).

Tamoxifen, a selective oestrogen receptor modulator (SERM), has been the standard adjuvant hormonal treatment for early-stage hormone receptor-positive breast cancer for more than two decades (Osborne, 1998). It has antagonistic effects on breast tissue, thereby inhibiting the growth of hormone-responsive tumours. Classically, tamoxifen has been considered to have oestrogenic effects on bone, and postmenopausal women typically experience an increase in BMD with tamoxifen therapy (Love *et al*, 1992; Kristensen *et al*, 1994; Powles *et al*, 1996). Conversely, tamoxifen therapy results in decreased BMD in premenopausal women, although the reason for this difference remains unclear (Powles *et al*, 1996; Sverrisdottir *et al*, 2004). One hypothesis is that tamoxifen exerts an effect more as an anti-oestrogen in bone in the premenopausal population because of the greater amount of circulating oestrogens compared with postmenopausal women (Powles *et al*, 1996).

Tamoxifen, a pro-drug, is converted into more active metabolites by cytochrome (CYP) P450 enzymes, primarily by CYP2D6 (Jin *et al*, 2005). *CYP2D6* is a highly polymorphic gene. Indeed, more than 80 different alleles have been identified, many of which confer a decreased or absent CYP2D6 activity. As has been previously shown in the cohort of patients described in this report, there is an association between decreased CYP2D6 activity and decreased serum concentrations of endoxifen, one of the main active metabolites of tamoxifen (Jin *et al*, 2005). Data suggest that women with the CYP2D6 poor metaboliser phenotype have worse breast cancer outcomes, and may have different tamoxifen-related toxicity profiles compared with women with a normal CYP2D6 activity (Goetz *et al*, 2005; Desta and Flockhart, 2007).

Tamoxifen acts via modulation of oestrogen receptors (ER). Single-nucleotide polymorphisms (SNPs) have been identified in both *ESR1* and *ESR2*, the genes that encode for ER-*α* and ER-*β*, respectively. These SNPs have been shown to be associated with breast cancer risk and breast cancer survival, as well as with serum lipid concentrations in healthy women (Herrington *et al*, 2002; Gold *et al*, 2004; Boyapati *et al*, 2005). In contrast, numerous studies have evaluated associations between BMD and ER genotypes in non-breast cancer patients (Willing *et al*, 1998; Ioannidis *et al*, 2004; Sowers *et al*, 2004; Gennari *et al*, 2007), but these results have been inconsistent. Few results have been reported that associate ER polymorphisms with a change in BMD in women treated with SERMs (Yoneda *et al*, 2002; Heilberg *et al*, 2005).

To further analyse the associations between genetic variants in *ESR1*, *ESR2*, and *CYP2D6* on BMD, we initiated a prospective study to evaluate the changes in BMD in both pre- and postmenopausal women who were recommended tamoxifen either as adjuvant therapy for newly diagnosed breast cancer or for chemoprevention. Baseline BMD and change in BMD with 1 year of therapy were tested for associations with polymorphisms in *ESR1, ESR2,* and *CYP2D6*.

## Materials and methods

### Subjects

Eligible patients were recruited into a prospective, observational, open-labelled, multicentre registry study conducted by the Consortium on Breast Cancer Pharmacogenomics (COBRA) from 2000 to 2006. Participating COBRA institutions included the Lombardi Comprehensive Cancer Center at Georgetown University Medical Center, Washington, DC; the Indiana University Cancer Center, Indianapolis, Indiana; and the Breast Oncology Program at the University of Michigan Comprehensive Cancer Center, Ann Arbor, Michigan. The analysis presented in this report is a secondary aim of the main study, and was specifically designed to test relationships between polymorphisms in the genes that encode ER and CYP2D6 and change in BMD after 1 year of tamoxifen therapy. Results from this cohort regarding other genotypes and phenotypes have been previously reported (Jin *et al*, 2005, 2008; Borges *et al*, 2006; Henry *et al*, 2009). The study design for this trial has already been described in detail (Jin *et al*, 2005) and is listed on www.ClinicalTrials.gov (NCT0022893).

Pre- and postmenopausal women 18 years or older, who had oestrogen and/or progesterone receptor-positive breast cancer, were starting adjuvant treatment with tamoxifen, had a history of ductal carcinoma *in situ*, or were at high risk for breast cancer and starting tamoxifen for chemoprevention were eligible for the study. Women were ineligible if they were treated with hormone therapy other than tamoxifen or if they started tamoxifen therapy concurrently with either radiation therapy or chemotherapy. Other exclusion criteria included use of concomitant clonidine, bellergal, megestrol acetate, or chronic corticosteroids (previous intermittent use of steroids during adjuvant chemotherapy was permitted). Patients were excluded if they were pregnant or lactating. No restrictions were placed on the use of bisphosphonates for trial enrolment, although subjects taking bisphosphonates were excluded from the BMD analyses, as described below. Use of supplemental calcium and vitamin D was encouraged. The protocol was approved by the institutional review boards of all three participating study sites. All enrolled patients provided written informed consent.

### Study design

Participants with invasive breast cancer or ductal carcinoma *in situ* were enrolled in the trial after completion of primary surgical resection of breast cancer, and after any indicated adjuvant chemotherapy and/or radiation therapy. Before starting tamoxifen, a complete medical history, comprehensive list of medications, physical examination, and baseline laboratory studies were obtained from each patient. Menopausal status was determined on the basis of self-reported menstrual history. Women who were 60 years or older, who were amenorrhoeic for 12 months before enrolment, or had undergone previous bilateral oophorectomy were considered postmenopausal. Women with regular menses before adjuvant chemotherapy or tamoxifen were considered premenopausal. All other patients were considered perimenopausal. Patients were followed up in the outpatient clinic 1, 4, 8, and 12 months after the initiation of treatment with tamoxifen at 20 mg per day. During each follow-up visit, changes in medical history and medication use were reviewed.

Blood samples (∼10 ml) were collected at baseline in heparinised Vacutainer tubes (Becton-Dickinson, Franklin Lakes, NJ, USA) for plasma isolation and genomic DNA extraction for genotyping analysis. Plasma was separated within 1 h of blood collection by centrifugation at 2060 **g**. All samples (plasma and whole blood) were then transferred to cryogenic vials (Corning, Cambridge, MA, USA) and stored at −80 °C pending analysis.

Standard dual-energy X-ray absorptiometry (DXA) scans were performed to measure BMD immediately before initiation of tamoxifen and at the completion of 1 year of therapy. The BMD values (calculated in grams per square centimetre) of the left femoral neck (hip) and total lumbar spine were measured at the University of Michigan using a Lunar DPX-L bone densitometer (Madison, WI, USA) and at Indiana University and Georgetown University using Hologic 4500 bone densitometers (Bedford, MA, USA). Baseline and 12-month DXA assessments were performed on the same bone densitometer for all but 17% of subjects at a single study site (14% of the total population). The BMD data set can be found on http://www.pharmgkb.org with the accession ID PS207749.

### Genotyping analysis

Genotyping for two ER-α (*ESR1*) SNPs (*Xba*I=rs 9340799 and *Pvu*II=rs 2234693) and two ER-*β* (*ESR2*) SNPs (ESR2_01=rs 1256049 and ESR2_02=rs 4986938) was performed in duplicate by Taqman assays as previously described by the NCI CGAP project (snp500cancer.nci.nih.gov). Amplification and analysis were performed using the iCycler real-time thermocycler (Bio-Rad, Hercules, CA, USA). Genotyping for *CYP2D6* variants was performed as previously described (Borges *et al*, 2006). Genotyping data can be found on the Pharmacogenetics and Pharmacogenomics Knowledge Base website (http://www.pharmgkb.org) with accession IDs *ESR1* (PS204992 and PS204997), *ESR2* (PS205000 and PS203537, PS203538 and PS204999), and *CYP2D6* (PS204849, PS204850, PS204858, PS204859, PS204873, PS204874, PS204875, PS204901, PS204991, and PS204996).

### Statistical analysis

The primary end point for this study was to test the association between menopausal status, previous chemotherapy, genotype, and mean percentage change in BMD over 12 months. Because of similarities in bone biology in peri- and postmenopausal women, data from peri- and postmenopausal patients were combined and compared with data from premenopausal patients in this analysis (Steinberg *et al*, 1989; Slemenda *et al*, 1996; Finkelstein *et al*, 2008). Analyses were performed on the basis of intention-to-treat, regardless of compliance with the study drug. Univariate analysis was conducted for all BMD outcomes (hip and lumbar spine BMD at baseline and the percentage change in BMD from months 0 to 12) to obtain descriptive statistics of variables and study their underlying distributions. Association between menopausal status, previous chemotherapy, and other categorical variables with BMD outcomes was examined using *t*-tests or ANOVA with *post hoc* pairwise comparisons. A general linear model was used to adjust for age and centre and to assess the interactions between menopausal status and previous chemotherapy, and was performed using the SAS program (Cary, NC, USA) (PROC GLM, SAS v9.1.3). Differences in each BMD outcome among genotypes, including age- and centre-adjusted analysis, were also examined using a general linear model. For *post hoc* comparisons, we compared the adjusted means between all pairs of three genotypes while controlling for overall alpha using the Tukey–Kramer method. For all analyses, a *P*-value of <0.05 was considered to be statistically significant.

Our data suggest that the lumbar BMD percentage change from baseline to 1 year after tamoxifen has an s.d. of 0.06; and 218 samples provide us with 88% power to detect the 1.3% observed lumbar BMD change. However, we only have 30% power to detect the 0.4% observed hip BMD change, which has an s.d. of 0.04. The type I error is set at the 5% level for each test, and they are based on a one-sample *t*-test. This pharmacogenetics substudy, which was a secondary aim of the overall study, was designed to test the associations between polymorphisms in candidate genes and change in BMD with 1 year of tamoxifen therapy. Because this analysis was not the primary aim, however, the power to detect our observed genetic effect on BMD changes in tamoxifen-treated patients was <20%.

The *ESR1* haplotypes were constructed from *ESR1 Pvu*II and *ESR1 Xba*I SNPs using the PHASE2 online software (http://www.stat.washington.edu/ stephens/software.html). The *ESR2* haplotypes were also constructed but were few because of the low frequency of *ESR2_01* variants. Haplotype associations with baseline BMD and percentage change in BMD were tested with a generalised estimating equation approach through its online implementation (http://www.mayo.edu/statgene) (Schaid *et al*, 2002).

## Results

### Subjects

Of the 297 patients who enrolled in the clinical trial, 21 were excluded from BMD analyses because of concomitant bisphosphonate use during the 1-year study duration (*n*=17, 5.7%), significantly outlying values of percentage BMD change (*n*=2), use of raloxifene at the time of study enrolment (*n*=1), and failure to initiate protocol-directed treatment (*n*=1) (for CONSORT diagram see Supplementary Figure 1). All 276 remaining patients were included in the baseline BMD analyses. Of these 276 patients, 11 did not have any baseline DXA measurements, 3 did not have baseline lumbar spine DXA measurements, and 9 did not have baseline hip DXA measurements. Therefore, 95% of analysed subjects had baseline lumbar spine DXA measurements, and 93% had baseline hip DXA measurements. Baseline characteristics of the analysed cohort are described in Table 1. Characteristics of the 21 excluded patients were similar to those of the analysed cohort, except for the fact that they were significantly older (mean age 63.2, *P*<0.0001) and all were postmenopausal.

In total, 58 patients did not have matched baseline and 12-month DXA assessments for the following reasons: missing baseline and/or 12-month measurements at either the hip or the spine (*n*=17), premature discontinuation of study participation for disease progression (*n*=1), toxicity (*n*=18), a switch to aromatase inhibitor (*n*=2), relocation (*n*=2), non-compliance or withdrawal of consent (*n*=8), and unknown (*n*=10). Data on hip BMD were missing on seven patients at one or both time points either because of machine malfunction or previous bilateral hip replacement. Thus, a total of 211 patients (76% of analysed cohort) had both baseline and 12-month DXA assessment at the hip, and 218 patients (79% of analysed cohort) had both DXA assessments at the lumbar spine. These subjects are included in the analyses evaluating percentage change in BMD with 1 year of tamoxifen therapy. There were no differences between the baseline characteristics of patients with and without 12-month DXA assessments.

Polymorphisms in genes encoding ER-*α* (*ESR1 Pvu*II and *Xba*I) and ER-*β* (*ESR2*_01 and _02) were determined for 260 patients (Table 2). No genotypes were determined for 16 patients because of lack of sufficient samples for analysis. A minority of samples were unable to be genotyped for every gene, as indicated in Table 2. All genotypes were in Hardy–Weinberg equilibrium. We observed three possible *ESR1* haplotypes on the basis of the *ESR1 Pvu*II and *Xba*I genotype: C-A 14.2%, C-G 35.0%, and T-A 50.8%. The three *ESR2* haplotypes based on *ESR2*_01 and _02 genotypes were A-G 3.8%, G-A 35.5%, and G-G 60.8%.

### Baseline BMD

Mean baseline BMD for all patients eligible for analysis divided by their menopausal and chemotherapy status is listed in Table 3, and the mean T scores are provided in Supplementary Table 1. Lumbar spine but not hip BMD was statistically significantly greater in premenopausal compared with postmenopausal women (*P*=0.032). Similar results were noted for the baseline T score at the lumbar spine (premenopausal +0.3, postmenopausal −0.3; *P*=0.003). No statistically significant differences in either the BMD or T score were noted between subjects treated and not treated with chemotherapy.

### Percentage change in BMD with 1 year of tamoxifen

The percentage change in BMD was calculated for each patient with matched baseline and 12-month BMD determinations. The percentage change in hip and lumbar spine BMD for all patients in aggregate, as well as for patients divided by chemotherapy and/or menopausal status, is presented in Tables 3 and 4, respectively. The average change in T score between baseline and 12 months for patients divided by chemotherapy or menopausal status is given in Supplementary Table 1.

Similar to previously reported results, postmenopausal women had an increase in BMD at the hip (+0.8%) and stability of BMD at the lumbar spine (−0.1%) with 1 year of tamoxifen therapy (Love *et al*, 1992; Kristensen *et al*, 1994; Powles *et al*, 1996). Consistent with earlier reports, BMD decreased at both the hip (−0.5%) and lumbar spine (−2.4%) in premenopausal women (Powles *et al*, 1996; Sverrisdottir *et al*, 2004). The percentage change in BMD at the lumbar spine was statistically significantly different in premenopausal compared with postmenopausal women (*P*=0.002), but only a trend was noted at the hip (*P*=0.065).

Patients who had received chemotherapy before initiation of tamoxifen therapy had an increase in both lumbar spine (+0.5%) and hip (+1.3%) BMD, whereas those who did not receive chemotherapy had a decrease in BMD at both sites (−2.2 and −0.5%, respectively), differences that were statistically significant (lumbar spine: *P*<0.001, hip: *P*=0.007). The difference in the percentage change in BMD between chemotherapy-treated and -untreated subjects remained when patients were subdivided by menopausal status (Table 4). Differences were statistically significant for postmenopausal women at both the lumbar spine (+1.4 *vs* −1.5% *P*<0.001) and hip (+1.8 *vs* −0.1% *P*=0.025), and for premenopausal women at the hip (−1.1 *vs* −3.6% *P*=0.030) but not at the lumbar spine.

### Effect of ER polymorphisms on baseline BMD

We evaluated the associations between two *ESR1* (*Pvu*II and *Xba*I) and two *ESR2* (*ESR2*_01 and *ESR2*_02) polymorphisms and baseline BMD. No association was noted between baseline BMD in the overall patient cohort or in any patient subgroup for the four polymorphisms studied (Supplementary Tables 2–5).

### Effect of ER polymorphisms on percentage change in BMD

The same *ESR1* and *ESR2* polymorphisms were evaluated for association with percentage change in BMD at the lumbar spine and hip. No statistically significant associations were noted between percentage change in BMD and single genotypes in any of the patient subgroups (Supplementary Tables 6–9). Similarly, no statistically significant association was observed between *ESR1* haplotypes and percentage change in BMD.

### Effect of CYP2D6 genotype on BMD

Because we previously showed that serum concentrations of endoxifen, a key active metabolite of tamoxifen, are lower in patients with *CYP2D6* variants associated with decreased or absent CYP2D6 activity (Jin *et al*, 2005; Borges *et al*, 2006), we evaluated whether there was an association between the *CYP2D6* genotype and percentage change in BMD with 1 year of tamoxifen therapy. We did not observe a statistically significant association between the *CYP2D6* genotype and either baseline BMD or percentage change in BMD in tamoxifen-treated patients (data not shown).

## Discussion

In this prospective clinical trial of pre- and postmenopausal women initiating therapy with tamoxifen, the subjects underwent BMD assessment at baseline and at 1 year to determine the effects of tamoxifen therapy on change in BMD. Approximately half of the patients in this trial had previously received chemotherapy, which was fairly evenly divided between the menopausal groups. Our data regarding the general effects of tamoxifen as a SERM are consistent with previously reported results in which premenopausal women have a decrease in BMD with tamoxifen therapy that is greater at the lumbar spine than at the hip (Powles *et al*, 1996; Sverrisdottir *et al*, 2004), whereas postmenopausal women experience an increase in BMD that is greater at the hip than at the lumbar spine (Love *et al*, 1992; Powles *et al*, 1996).

In this study, baseline BMD was similar for patients treated with or without chemotherapy. However, when evaluating the change with tamoxifen therapy, our data suggest that tamoxifen caused a greater increase in BMD in women who received previous chemotherapy compared with those who did not receive chemotherapy, regardless of menopausal status. This effect does not seem to have been previously reported in literature.

Although we do not have an explanation for this finding, one possible hypothesis is that chemotherapy-induced alterations in either bone metabolism or ovarian function lead to augmentation of the oestrogenic effects of tamoxifen on bone. Premenopausal women who develop chemotherapy-induced amenorrhoea have been shown to have rapid bone loss compared with those who retain menstrual function (Saarto *et al*, 1997; Shapiro *et al*, 2001; Fuleihan Gel *et al*, 2005). Some reports have shown no effect of post-chemotherapy tamoxifen on BMD (Shapiro *et al*, 2001). In contrast, others have shown that premenopausal women treated with chemotherapy, followed by tamoxifen, who continued to menstruate 3 years after chemotherapy, have increased bone loss during tamoxifen therapy compared with controls; in contrast, those who develop chemotherapy-induced amenorrhoea have less bone loss during tamoxifen therapy (Vehmanen *et al*, 2006). Studies in men of a different SERM, raloxifene, also found an association between increased bone resorption and higher serum oestrogen levels, and suppressed bone turnover and lower oestrogen levels (Doran *et al*, 2001; Uebelhart *et al*, 2004). Therefore, only a subset of data in literature supports our hypothesis. In our patient cohort, we do not have information regarding recovery of ovarian function and we lack sufficient serum samples for measurement of oestradiol concentrations to assess menopausal status. However, the increase in BMD in chemotherapy-treated patients was noted in both pre- and postmenopausal women, suggesting that ovarian function recovery was not the primary cause of the differential effect of tamoxifen on BMD in chemotherapy-treated *vs* untreated patients. It is possible that these findings are an artefact of the small sample size, although the high level of statistical significance for the overall population suggests that the effect may be clinically significant. Given these potential confounding factors, a confirmation of these findings in another sample set is warranted.

In postmenopausal women who did not receive chemotherapy, we did not find as great an increase in BMD with tamoxifen therapy as has previously been reported (Powles *et al*, 1996). One possible explanation for this discrepancy is differences in the earlier usage of hormone replacement therapy, although we cannot confirm this hypothesis because these data were not collected in our patient cohort. Another potential confounder is the substantially higher body mass index in our patient cohort (28.8 kg m^−2^) compared with that reported by Powles *et al* (1996)(25.2 kg m^−2^). Finally, a third potential source of bias is the replacement of a DXA machine at one institution during the conduct of this study. However, the effect of this change on the outcome of the study is likely to be minimal, as all subjects at that institution had their baseline DXA scans performed on the same machine, and only 17% had their baseline and 12-month DXA assessments performed on different machines. In addition, this may more closely reflect standard clinical practice, in which patients are unlikely to undergo serial bone density assessment on the same machine. Although bone turnover markers could be assessed to confirm the changes noted on DXA scanning, we unfortunately do not have sufficient remaining serum or urine samples for this evaluation.

The main objective of this analysis was to correlate the influence of inherited polymorphisms in the genes that encode for ER (*ESR1* and *ESR2*) on baseline BMD and change in BMD with tamoxifen therapy. However, no associations were detected between any of the four polymorphisms evaluated and either baseline BMD or change in BMD with tamoxifen therapy, or within *ESR1* or *ESR2* haplotypes. This finding is consistent with an earlier report of tamoxifen therapy in postmenopausal Japanese women, in whom no association was noted between change in BMD and either of the two *ESR1* genotypes (Yoneda *et al*, 2002). We also found no association between percentage change in BMD and genotype variants in CYP2D6, a key enzyme responsible for the conversion of tamoxifen into an active metabolite.

The paucity of statistically significant associations between ER genotypes and change in BMD with tamoxifen therapy in this study may reflect a true lack of association, or may be because of the heterogeneity and small sample size of the cohort. More than 1000 SNPs have been identified in *ESR1*, but the functional consequence of each SNP has not been characterised. Therefore, we chose to analyse the effects of SNPs that were previously extensively evaluated and were shown to have functional importance. However, as only four genotypes were evaluated in this analysis, a real effect may have been missed. Additional SNPs in the genes encoding ER, including those identified in recent genome-wide association studies (Richards *et al*, 2008; Styrkarsdottir *et al*, 2008), will be evaluated in the future when more data are available regarding the clinical effect of these mutations. In addition, an evaluation of associations between SNPs in genes encoding ER co-activators and corepressors and change in BMD is currently underway (Richter *et al*, 2007).

Although we did not observe any statistically significant associations between genetic variability and change in BMD, our power to detect small associations was quite low (<20%). Thus, it is possible that small single gene effects are present, but were not detected. In addition, because of the complexity of gene–gene interactions, it is possible that no single genotype–phenotype association is sufficiently strong to be evident, but rather a combination of genotypes may be required for a meaningful effect. Given the difficulties with multiple gene comparisons, a cohort of this size would be insufficient to arrive at meaningful conclusions if the effect sizes are small. Although it is possible that a comprehensive haplotype-tagging approach may engender useful associations, it is clear that no large effect due to these germline variants, which have previously been shown to influence BMD, is evident in this study. It is possible that a pathway approach involving an examination of genetic variants in genes that code for other elements within the oestrogen signalling pathways may reveal valuable mechanistic and predictive data, and that a genome-wide variant analysis might also be useful, but such examinations are beyond the scope of this study.

In summary, our data are consistent with previously reported data on the change in BMD with tamoxifen therapy in pre- and postmenopausal women. No inherited gene variants were found to be statistically significantly associated with baseline or change in BMD during tamoxifen therapy. The finding that previous chemotherapy may influence BMD response to tamoxifen is provocative and worthy of further study.

## Figures and Tables

**Table 1:** Demographic information of 276 patients included in BMD analyses

**Characteristic**	**All subjects^a^ (*n*=276)**	**Premenopausal (*n*=94)**	**Postmenopausal (*n*=180)**
Mean age (s.d.)	51.9 (10.0)	43.7 (6.5)	56.2 (8.7)
			
*Menopausal status* (%)		94 (34.3)	180 (65.7)
Perimenopausal (%)			37 (13.5)
Postmenopausal (%)			143 (52.2)
			
Weight in kg (s.d.)	76.1 (16.5)	73.5 (17.4)	77.5 (15.9)
Body mass index^b^ (s.d.)	28.2 (6.3)	27.1 (6.5)	28.8 (6.2)
			
*Chemotherapy* c			
Yes (%)	132 (48)	44 (16.1)	88 (32.1)
No (%)	143 (52)	50 (18.2)	92 (33.6)

Abbreviation: BMD=bone mineral density.

a Menopausal data missing for two patients.

b Height missing for 37 patients.

c Data missing for one patient.

**Table 2:** Characteristics of the analysed SNPs in *ESR1* and *ESR2* for subjects included in this analysis

**SNP**	**dbSNP**	**Location within gene**	**% Allele frequency**	**Genotype frequency**	**Subjects from this analysis**
			C 0.52	CC 0.245	CC 0.233 (*n*=60)
*ESR1* *Pvu*II^a^	rs 2234693	IVS1-397T>C	T 0.48	CT 0.549	CT 0.512 (*n*=132)
				TT 0.206	TT 0.256 (*n*=66)
					
			A 0.64	AA 0.462	AA 0.430 (*n*=110)
*ESR1* *Xba*I^b^	rs 9340799	IVS1-351A>G	G 0.36	AG 0.355	AG 0.453 (*n*=116)
				GG 0.183	GG 0.117 (*n*=30)
					
			A 0.108	AA 0.020	AA 0.008 (*n*=2)
*ESR2*_01^c^	rs 1256049	Ex6+32G>A	G 0.892	AG 0.176	AG 0.062 (*n*=16)
				GG 0.804	GG 0.931 (*n*=242)
					
*ESR2*_02^d^	rs 4986938	38 bp 3′ of STP A>G (3′UTR)	A 0.260	AA 0.088	AA 0.136 (*n*=35)
			G 0.740	AG 0.343	AG 0.440 (*n*=113)
				GG 0.569	GG 0.424 (*n*=109)

Abbreviation: dbSNP=single-nucleotide polymorphism database. Allele and genotype frequency data from SNP500Cancer controls (http://snp500cancer.nci.nih.gov/home_1.cfm).

a Data missing for 18 patients.

b Data missing for 20 patients.

c Data missing for 16 patients.

d Data missing for 19 patients.

**Table 3:** Baseline BMD and change in BMD with 12-month tamoxifen therapy

**Time point**	**All patients**	**Premenopausal**	**Postmenopausal**	**Previous chemotherapy**	**No chemotherapy**
*Lumbar spine*					
Baseline (g cm^−2^)	1.14 (1.12 to 1.16)	1.17 (1.14 to 1.21)^a^	1.13 (1.10 to 1.15)^a^	1.14 (1.11 to 1.16)	1.15 (1.12 to 1.18)
	*n*=262	*n*=91	*n*=171	*n*=123	*n*=139
% Change at 12 months after tamoxifen	−0.9 (−1.6 to −0.2)	−2.4 (−3.6 to −1.2)^b^	−0.1 (−1.0 to 0.7)^b^	+0.5 (−0.5 to 1.5)^d^	−2.2 (−3.2 to−1.3)^d^
	*n*=218	*n*=77	*n*=141	*n*=105	*n*=113
					
*Hip*					
Baseline (g cm^−2^)	0.99 (0.97 to 1.01)	1.00 (0.97 to 1.03)	0.98 (0.96 to 1.01)	0.98 (0.96 to 1.00)	1.00 (0.97 to 1.02)
	*n*=256	*n*=90	*n*=166	*n*=120	*n*=136
% Change at 12 months after tamoxifen	0.4 (−0.3 to 1.0)	−0.5 (−1.5 to 0.6)^c^	+0.9 (0.0 to 1.7)^c^	+1.3 (0.4 to 2.2)^e^	−0.5 (−1.5 to 0.4)^e^
	*n*=211	*n*=78	*n*=133	*n*=103	*n*=108

Abbreviation: BMD=bone mineral density.

For baseline DXA measurements, the 95% confidence interval of mean BMD is given for all patients in each subgroup. For percentage change at 12 months, 95% confidence interval of mean percentage change is given for those patients in each subgroup with both baseline and 12-month measurements.

*P*-values signify comparisons between two means with the same letter.

^a^*P*=0.032.

^b,d,e^*P*<0.01.

^c^*P*=0.065.

**Table 4:** Baseline and percentage change in BMD by menopausal and chemotherapy status

	**Lumbar spine**		**Hip**	
**Chemo**	**Premenopausal**	**Postmenopausal**	**Premenopausal**	**Postmenopausal**
*Baseline* (*g* *cm*^−*2*^)				
Yes	1.166 (1.124 to 1.208)	1.119 (1.083 to 1.156)	1.001 (0.966 to 1.035)	0.968 (0.938 to 0.998)
	*n*=43	*n*=80	*n*=42	*n*=78
No	1.181 (1.129 to 1.235)	1.134 (1.0978 to 1.170)	1.002 (0.954 to 1.050)	0.996 (0.966 to 1.023)
	*n*=48	*n*=91	*n*=48	*n*=88
				
*Percentage change*				
Yes	−1.1 (−2.9 to 0.6)^*^	+1.4 (0.2 to 2.5)^*^	+0.4 (−1.2 to 2.1)	+1.8 (0.7 to 3.0)^*^^*^
	*n*=38	*n*=67	*n*=38	*n*=65
No	−3.6 (−5.2 to −2.0)^*^	−1.5 (−2.7 to −0.3)^*^	−1.3 (−2.7 to 0.1)	−0.1 (−1.3 to 1.2)^*^^*^
	*n*=39	*n*=74	*n*=40	*n*=68

Abbreviation: BMD=bone mineral density.

For baseline DXA measurements, 95% confidence interval of mean BMD is given for all patients in each subgroup. For percentage change at 12 months, 95% confidence interval of mean percentage change is given for those patients in each subgroup with both baseline and 12-month measurements.

*P*-values signify comparisons between two means with the same symbol.

^*^P<0.05.

^*^^*^P<0.01.
