# Supplementary material for: Lack of association between oestrogen receptor polymorphisms and change in bone mineral density with tamoxifen therapy
Source: Br J Cancer. 2009 Dec 1;102(2):294–300. doi: 10.1038/sj.bjc.6605460 (PMC2816645; doi:10.1038/sj.bjc.6605460)
Supplement: Supplementary Information [file 6605460x1.doc]

Supplemental Figure 1: Consort diagram demonstrating flow of patients through the study. LS = lumbar spine.

**Table 1. Baseline T score and change in T score with 12 mo tamoxifen therapy.** For baseline T score, 95 % confidence interval of mean T score is given for all patients in each subgroup, and standard deviations are listed in parentheses. For average change in T score between baseline and 12 months, 95 % confidence interval of mean change is given for those patients in each subgroup with both baseline and 12 month measurements, and standard errors are listed in parentheses. P values signify comparisons between 2 means with the same symbol.

No

Time point All patients Premenopausal Postmenopausal Chemotherapy chemotherapy

**Lumbar spine**

Baseline -0.1 (-0.2, 0.1) 0.3 (0.0, 0.5)^*^ -0.3 (-0.5, 0.0)^*^ -0.1 (-0.3, 0.1) -0.1 (-0.3, 0.2)

n=262 n=91 n=171 n=123 n=139

change at 12 mo -0.2 (-0.3, -0.1) -0.4 (-0.6, -0.2)^#^ 0.0 (-0.1, 0.1)^#^ -0.1 (-0.2, 0.1) -0.2 (-0.3, -0.1)

after tamoxifen n=218 n=77 n=141 n=105 n=113

**Hip**

Baseline 0.0 (-0.1, 0.2) 0.1 (-0.1, 0.4) 0.0 (-0.2, 0.1) -0.1 (-0.2, 0.1) 0.1 (-0.1, 0.3)

n=256 n=90 n=166 n=120 n=136

change at 12 mo 0.0 (-0.1, 0.0) -0.1 (-0.2, 0.0) 0.0 (-0.1, 0.1) 0.1 (0.0, 0.1)^§^ -0.1 (-0.2, 0.0) ^§^

after tamoxifen n=211 n=78 n=133 n=103 n=108

^*, §^ p<0.01

^#^ p<0.001

Table 2. **Effect of ER polymorphisms on baseline BMD in premenopausal women who did not receive chemotherapy**

|  |  | Hip | | | L spine | | |
| --- | --- | --- | --- | --- | --- | --- | --- |
| Gene | SNP | # pts | LS mean | P value gene-dose effect | # pts | LS mean | P value for gene-dose effect |
| ESR1 *PvuII* | CC | 13 | 0.967 | 0.353 | 13 | 1.103 | 0.147 |
|  | CT | 23 | 0.978 |  | 23 | 1.147 |  |
|  | TT | 11 | 0.896 |  | 11 | 1.037 |  |
| ESR1 *XbaI* | AA | 20 | 0.933 | 0.350 | 20 | 1.073 | 0.323 |
|  | AG | 22 | 0.959 |  | 22 | 1.129 |  |
|  | GG | 5 | 1.0490 |  | 3 | 1.174 |  |
| ESR2_01 | AG | 3 | 0.892 | 0.649 | 3 | 1.074 | 0.820 |
|  | GG | 43 | 0.935 |  | 43 | 1.094 |  |
| ESR2_02 | AA | 6 | 1.000 | 0.482 | 6 | 1.138 | 0.720 |
|  | AG | 17 | 0.909 |  | 17 | 1.084 |  |
|  | GG | 23 | 0.929 |  | 23 | 1.084 |  |

Table 3. **Effect of ER polymorphisms on baseline BMD in premenopausal women who received chemotherapy**

|  |  | Hip | | | L spine | | |
| --- | --- | --- | --- | --- | --- | --- | --- |
| Gene | SNP | # pts | LS mean | P value gene-dose effect | # pts | LS mean | P value for gene-dose effect |
| ESR1 *PvuII* | CC | 9 | 0.943 | 0.250 | 9 | 1.018 | 0.019 |
|  | CT | 22 | 0.995 |  | 23 | 1.143 |  |
|  | TT | 7 | 0.938 |  | 7 | 1.062 |  |
| ESR1 *XbaI* | AA | 13 | 0.946 | 0.648 | 14 | 1.082 | 0.136 |
|  | AG | 21 | 0.975 |  | 21 | 1.126 |  |
|  | GG | 4 | 0.939 |  | 4 | 0.996 |  |
| ESR2_01 | AG | 1 | 1.018 | 0.816 | 2 | 1.111 | 0.918 |
|  | GG | 37 | 0.991 |  | 37 | 1.102 |  |
| ESR2_02 | AA | 5 | 0.989 | 0.685 | 5 | 1.059 | 0.592 |
|  | AG | 15 | 0.968 |  | 15 | 1.121 |  |
|  | GG | 18 | 1.004 |  | 19 | 1.101 |  |

Table 4. **Effect of ER polymorphisms on baseline BMD in postmenopausal women who did not receive chemotherapy**

|  |  | Hip | | | L spine | | |
| --- | --- | --- | --- | --- | --- | --- | --- |
| Gene | SNP | # pts | LS mean | P value gene-dose effect | # pts | LS mean | P value for gene-dose effect |
| ESR1 *PvuII* | CC | 18 | 0.909 | 0.636 | 19 | 1.009 | 0.676 |
|  | CT | 45 | 0.939 |  | 47 | 1.045 |  |
|  | TT | 22 | 0.952 |  | 22 | 1.052 |  |
| ESR1 *XbaI* | AA | 37 | 0.939 | 0.306 | 37 | 1.051 | 0.482 |
|  | AG | 37 | 0.904 |  | 39 | 1.036 |  |
|  | GG | 9 | 0.864 |  | 10 | 0.978 |  |
| ESR2_01 | AG | 7 | 1.008 | 0.172 | 7 | 1.052 | 0.814 |
|  | GG | 79 | 0.929 |  | 82 | 1.068 |  |
| ESR2_02 | AA | 11 | 0.971 | 0.579 | 12 | 1.067 | 0.843 |
|  | AG | 41 | 0.938 |  | 42 | 1.075 |  |
|  | GG | 32 | 0.919 |  | 33 | 1.052 |  |

Table 5. **Effect of ER polymorphisms on baseline BMD in postmenopausal women who received chemotherapy**

|  |  | Hip | | | L spine | | |
| --- | --- | --- | --- | --- | --- | --- | --- |
| Gene | SNP | # pts | LS mean | P value gene-dose effect | # pts | LS mean | P value for gene-dose effect |
| ESR1 *PvuII* | CC | 18 | 0.963 | 0.436 | 18 | 1.081 | 0.635 |
|  | CT | 36 | 0.912 |  | 36 | 1.035 |  |
|  | TT | 20 | 0.927 |  | 22 | 1.057 |  |
| ESR1 *XbaI* | AA | 32 | 0.931 | 0.990 | 34 | 1.061 | 0.944 |
|  | AG | 32 | 0.936 |  | 32 | 1.057 |  |
|  | GG | 10 | 0.932 |  | 10 | 1.041 |  |
| ESR2_01 | AA | 2 | 0.971 | 0.925 | 2 | 0.985 | 0.799 |
|  | AG | 3 | 0.938 |  | 3 | 1.054 |  |
|  | GG | 71 | 0.932 |  | 73 | 1.067 |  |
| ESR2_02 | AA | 10 | 0.919 | 0.855 | 10 | 1.092 | 0.815 |
|  | AG | 33 | 0.948 |  | 35 | 1.062 |  |
|  | GG | 32 | 0.938 |  | 32 | 1.052 |  |

Table 6. **Effect of ER polymorphisms on percentage change in BMD in premenopausal women who did not receive chemotherapy**

|  |  | Hip | | | L spine | | |
| --- | --- | --- | --- | --- | --- | --- | --- |
| Gene | SNP | # pts | % change LS mean | P value | # pts | % change LS mean | P value |
| ESR1 *PvuII* | CC | 11 | -0.1 | 0.215 | 10 | -3.5 | 0.636 |
|  | CT | 20 | 0.0 |  | 20 | -4.1 |  |
|  | TT | 8 | +2.0 |  | 8 | -2.0 |  |
| ESR1 *XbaI* | AA | 16 | +0.3 | 0.195 | 16 | -3.4 | 0.992 |
|  | AG | 19 | -0.3 |  | 19 | -3.6 |  |
|  | GG | 4 | -3.2 |  | 3 | -3.5 |  |
| ESR2_01 | AG | 3 | -0.8 | 0.480 | 3 | -4.5 | 0.684 |
|  | GG | 35 | +0.8 |  | 34 | -3.2 |  |
| ESR2_02 | AA | 4 | -0.2 | 0.653 | 4 | -1.8 | 0.285 |
|  | AG | 13 | +1.5 |  | 13 | -1.9 |  |
|  | GG | 21 | +0.5 |  | 20 | -4.6 |  |

Table 7. **Effect of ER polymorphisms on percentage change in BMD in premenopausal women who received chemotherapy**

|  |  | Hip | | | L spine | | |
| --- | --- | --- | --- | --- | --- | --- | --- |
| Gene | SNP | # pts | % change LS mean | P value | # pts | % change LS mean | P value |
| ESR1 *PvuII* | CC | 9 | +3.2 | 0.716 | 9 | -0.1 | 0.539 |
|  | CT | 18 | +1.6 |  | 18 | -1.8 |  |
|  | TT | 7 | +1.4 |  | 7 | -3.3 |  |
| ESR1 *XbaI* | AA | 12 | +1.9 | 0.562 | 12 | -2.6 | 0.605 |
|  | AG | 18 | +2.0 |  | 18 | -1.1 |  |
|  | GG | 4 | -1.1 |  | 4 | +0.4 |  |
| ESR2_01 | AG | 0 | n/a | n/a | 0 | n/a | n/a |
|  | GG | 34 | +1.0 |  | 34 | -1.6 |  |
| ESR2_02 | AA | 5 | -3.4 | 0.106 | 5 | -5.0 | 0.328 |
|  | AG | 13 | +2.1 |  | 13 | -0.7 |  |
|  | GG | 17 | +1.5 |  | 17 | -1.3 |  |

Table 8. **Effect of ER polymorphisms on percentage change in BMD in postmenopausal women who did not receive chemotherapy**

|  |  | Hip | | | L spine | | |
| --- | --- | --- | --- | --- | --- | --- | --- |
| Gene | SNP | # pts | % change LS mean | P value | # pts | % change LS mean | P value |
| ESR1 *PvuII* | CC | 15 | +2.4 | 0.708 | 16 | -3.0 | 0.894 |
|  | CT | 32 | +3.6 |  | 36 | -2.4 |  |
|  | TT | 16 | +2.9 |  | 17 | -3.1 |  |
| ESR1 *XbaI* | AA | 27 | +3.1 | 0.751 | 28 | -2.7 | 0.961 |
|  | AG | 28 | +3.4 |  | 32 | -2.9 |  |
|  | GG | 7 | +1.9 |  | 8 | -3.2 |  |
| ESR2_01 | AG | 6 | +2.3 | 0.256 | 6 | -3.2 | 0.668 |
|  | GG | 58 | +4.5 |  | 64 | -2.3 |  |
| ESR2_02 | AA | 8 | +5.4 | 0.709 | 10 | -3.7 | 0.481 |
|  | AG | 30 | +4.1 |  | 32 | -2.1 |  |
|  | GG | 24 | +3.8 |  | 26 | -1.3 |  |

Table 9. **Effect of ER polymorphisms on percentage change in BMD in postmenopausal women who received chemotherapy**

|  |  | Hip | | | L spine | | |
| --- | --- | --- | --- | --- | --- | --- | --- |
| Gene | SNP | # pts | % change LS mean | P value | # pts | % change LS mean | P value |
| ESR1 *PvuII* | CC | 14 | +3.6 | 0.592 | 14 | -0.2 | 0.572 |
|  | CT | 27 | +2.9 |  | 27 | +1.1 |  |
|  | TT | 18 | +2.0 |  | 20 | +1.7 |  |
| ESR1 *XbaI* | AA | 27 | +2.5 | 0.694 | 29 | +1.0 | 0.790 |
|  | AG | 25 | +2.7 |  | 25 | +0.2 |  |
|  | GG | 7 | +4.1 |  | 7 | +1.9 |  |
| ESR2_01 | AA | 2 | +4.8 | 0.135 | 2 | +2.9 | 0.852 |
|  | AG | 2 | -3.8 |  | 2 | +0.4 |  |
|  | GG | 58 | +2.8 |  | 60 | +0.9 |  |
| ESR2_02 | AA | 9 | +2.9 | 0.380 | 9 | -0.1 | 0.676 |
|  | AG | 28 | +1.3 |  | 29 | +1.0 |  |
|  | GG | 24 | +3.1 |  | 25 | +1.6 |  |
